# Management of diabetes mellitus in individuals with chronic kidney disease: therapeutic perspectives and glycemic control

**DOI:** 10.6061/clinics/2016(01)08

**Published:** 2016-01

**Authors:** Carolina C R Betônico, Silvia M O Titan, Maria Lúcia C Correa-Giannella, Márcia Nery, Márcia Queiroz

**Affiliations:** I Universidade Oeste Paulista, Hospital Regional de Presidente Prudente, Divisão de Endocrinologia, Presidente Prudente/, SP, Brazil; II Faculdade de Medicina da Universidade de São Paulo, Departamento de Medicina Interna, Divisão de Nefrologia; III Laboratório de Endocrinologia Celular e Molecular (LIM-25); IV Divisão de Endocrinologia, São Paulo/, SP, Brazil

**Keywords:** Type 2 Diabetes Mellitus, Chronic Kidney Disease, Diabetic Kidney Disease, Renal Failure, Diabetes Treatment, Oral Antidiabetic Drugs

## Abstract

The purpose of this study was to evaluate the therapeutic options for diabetes treatment and their potential side effects, in addition to analyzing the risks and benefits of tight glycemic control in patients with diabetic kidney disease. For this review, a search was performed using several pre-defined keyword combinations and their equivalents: “diabetes kidney disease” and “renal failure” in combination with “diabetes treatment” and “oral antidiabetic drugs” or “oral hypoglycemic agents.” The search was performed in PubMed, Endocrine Abstracts and the Cochrane Library from January 1980 up to January 2015. Diabetes treatment in patients with diabetic kidney disease is challenging, in part because of progression of renal failure-related changes in insulin signaling, glucose transport and metabolism, favoring both hyperglycemic peaks and hypoglycemia. Additionally, the decline in renal function impairs the clearance and metabolism of antidiabetic agents and insulin, frequently requiring reassessment of prescriptions. The management of hyperglycemia in patients with diabetic kidney disease is even more difficult, requiring adjustment of antidiabetic agents and insulin doses. The health team responsible for the follow-up of these patients should be vigilant and prepared to make such changes; however, unfortunately, there are few guidelines addressing the nuances of the management of this specific population.

## INTRODUCTION

Diabetes mellitus is the leading cause of chronic kidney disease (CKD) and a major public health issue worldwide. Approximately 20–30% of patients with type 2 diabetes mellitus (T2DM) have renal impairment, classified as moderate-to-severe CKD (glomerular filtration rate (GFR) <60 mL/min/1.73 m^2^) 1. Unfortunately, the combination of diabetes and CKD is associated with increased morbidity and mortality, mainly due to increased cardiovascular risk 2.

Glycemic control in patients with kidney failure faces special challenges. According to the progression of CKD, changes in insulin signaling, glucose transport and metabolism are associated with accumulation of uremic toxins, inflammatory factors and oxidative stress, inducing insulin resistance and the response of target tissues to insulin signaling 3-5.

The management of hyperglycemia in patients with CKD is especially difficult, in part because of the treatment complexity and in part due to insufficient convincing data supporting the benefits of tight glycemic control in this subset of patients. Furthermore, inherent risks, including severe hypoglycemia and increased cardiovascular risk, should be considered when formulating therapeutic strategies 6-8.

The aim of this review article was to evaluate the therapeutic options for diabetes treatment and their potential side effects, in addition to analyzing the risks and benefits of tight glycemic control in patients with diabetic kidney disease (DKD).

For this review, a search was performed using several pre-defined keyword combinations and their equivalents: “diabetes kidney disease” and “renal failure” in combination with “diabetes treatment” and “oral antidiabetic drugs” (OADs) or “oral hypoglycemic agents.” The search was performed in PubMed, Endocrine Abstracts and the Cochrane Library from January 1980 up to January 2015. Only full-text manuscripts published in English were included in the study.

### Antidiabetes therapy: Current options

Traditionally, insulin has been considered the safe choice for treating diabetic patients with kidney injury. Recently, new oral drug options have become good potential alternatives and traditional medications used in diabetes treatment have had their prescriptions and dosages reviewed.

### Biguanide - Metformin

Usually, metformin is the initial pharmacological agent for type 2 diabetes treatment 8. This drug acts mainly by decreasing hepatic glucose production, increasing peripheral glucose uptake, improving glucose tolerance and lowering fasting and postprandial plasma glucose. The prescription of metformin is contraindicated in DKD because it undergoes renal excretion 9 and its most serious adverse effect is the development of lactic acidosis, although this is a very rare occurrence, with approximately 5 cases per 100,000 patient-years 9. A current United Kingdom (UK) guideline on the treatment of T2DM allows metformin use up to a GFR of 30 mL/min/1.73 m^2^, with dose reduction advised at 45 mL/min/1.73 m^2^ 10,11. In the USA, metformin is contraindicated for men with serum creatinine ≥1.5 mg/dL and for women with serum creatinine ≥1.4 mg/dL 8-12. New evidence from the literature suggests that patients with mild-to-moderate DKD face more benefits than risks while using metformin 13-15. In fact, the REduction of Atherothrombosis for Continued Health (REACH) Registry 2004 showed decreased mortality associated with metformin use, even in patients with moderate kidney disease 16,17. Nonetheless, the use of metformin is still avoided in patients with CKD stages 3–5 with other associated risk factors for lactic acidosis. However, in recent years, studies based on experimental and cell culture models have shown a potential renal protective effect for metformin. In these studies, metformin prevented glucose-induced oxidative stress in podocytes by inhibiting NAD(P)H oxidase; decreasing 8-hydroxydeoxyguanosine (8-OHdG), a supposed marker of total systemic oxidative stress and DNA damage *in vivo*; and also improving the free-radical defense system 18-20.

### Sulfonylureas

Sulfonylureas (SUs) are drugs that stimulate endogenous insulin secretion by pancreatic β cells. These drugs may potentially cause hypoglycemia, especially in association with high doses; omission or reduction of carbohydrate intake; alcohol abuse; hepatic dysfunction; heart failure; malnutrition; advanced age; and interactions with certain drugs that displace SUs from their plasma protein-binding sites 21 because one or more of their metabolites may accumulate, resulting in an increased risk of hypoglycemia 7. Several drugs are included in this class. Glipizide is metabolized by the liver into several inactive metabolites and its clearance and elimination half-life are not affected by a reduction in the estimated GFR (eGFR), so dose adjustments are not necessary in patients with CKD 22. Therefore, glipizide is the SU of choice in patients with CKD. Glibenclamide and glyburide are each metabolized by the liver and are eliminated equally in the bile and urine. Hypoglycemic episodes may be severe in patients with renal failure, and the drugs are contraindicated from stage 3 of CKD (eGFR <60 mL/min). Meanwhile, glimepiride is metabolized by the liver into two main metabolites, one of which has hypoglycemic activity. In patients with renal impairment, these metabolites can accumulate. Nevertheless, glimepiride is associated with less hypoglycemia compared with glyburide, although its use should be avoided in patients with a GFR of <60 mL/min 23. Finally, gliclazide has inactive metabolites that are eliminated mainly in the urine (80%) and presents a lower risk of severe hypoglycemia than glibenclamide and glimepiride do. This drug can be considered in renal impairment if appropriate attention is paid to the dose. However, use should be avoided if the GFR falls to <40 mL/min 7,21.

### Glinides

Both repaglinide and nateglinide belong to a class of glinides, which are Su-like agents that stimulate insulin secretion but are rapidly absorbed, with a short duration of action, thus contributing to reducing postprandial hyperglycemia. Nateglinide (but not repaglinide) is hepatically metabolized, with renal excretion of active metabolites that are retained in DKD, so it should be used with caution in patients with advanced renal injury 24. In contrast, repaglinide is considered a safe option until the GFR falls to <30 mL/min/1.73 m^2^ 25,26. In advanced renal disease, treatment with repaglinide should begin cautiously, with 0.5 mg daily, to avoid hypoglycemia.

### Alpha-glucosidase inhibitors

Alpha-glucosidase inhibitors reduce the rate of digestion and intestinal absorption of carbohydrates, resulting in mild reduction of glycated hemoglobin (A1C). Acarbose is metabolized nearly completely within the gastrointestinal tract, so less than 2% of an oral dose is recovered as the active drug or its metabolites in the urine. The other drug in this class, or miglitol, is absorbed systemically and excreted unchanged in the urine. Given their modest efficacy in glycemic control and the lack of long-term trials in patients with kidney disease, these medications should be avoided in CKD stages 4 and 5 27,28.

### Glitazones

Pioglitazone is a thiazolidinedione (TZD) antidiabetic agent that is a potent and highly selective agonist of peroxisome proliferator-activated receptor-gamma (PPARγ). PPAR receptors are found in tissues important for insulin action, such as the adipose tissue, skeletal muscle, and liver. Activation of PPARγ nuclear receptors modulates the transcription of a number of insulin-responsive genes involved in the control of glucose and lipid metabolism. The pharmacokinetic profile of pioglitazone was found to be similar between healthy subjects and patients with moderately or severely impaired renal function who did not require dialysis 29. *Post hoc* analysis from the PROspective pioglitAzone Clinical Trial In macroVascular Events (PROactive) identified a greater decline in the eGFR with pioglitazone (between-group difference of 0.8 mL/min per 1.73 m(2)/yr) than with placebo 30. However, these medications may cause fluid retention and thus should be used with caution in patients with heart failure (HF) as well as in those with CKD and a significant decrease in the GFR 28.

### Dipeptidyl peptidase-4 inhibitors

Dipeptidyl peptidase-4 (DPP-4) inhibitors prevent degradation of incretin hormones by DPP-4, enhancing glucose-dependent insulin secretion. There are five available DPP-4 inhibitors, also known as “gliptins” (sitagliptin, vildagliptin, saxagliptin, linagliptin and alogliptin), and despite their common mechanism of action, these agents have structural heterogeneity that translates into different pharmacological properties and different metabolism and excretion pathways 31. Sitagliptin is mostly eliminated unchanged in the urine and can be used with appropriate dose reduction in all chronic kidney stages. The usual dose of 100 mg once per day should be adjusted to 50 mg/day for patients with moderate renal impairment. In severe renal impairment (creatinine clearance <30 mL/min) or end-stage renal disease (ESRD) requiring dialysis, the dose is further reduced to 25 mg once daily 32. Vildagliptin is metabolized, mostly in the kidneys, into inactive metabolites. Its main route of elimination is hydrolysis by multiple tissues or organs and approximately 25% of the drug is excreted unchanged by the kidneys. In type 2 diabetes patients and patients with moderate-to-severe CKD, dose reductions for vildagliptin are required, which means a reduction by half (to 50 mg/day) for both moderate and severe CKD 31-33. Saxagliptin is metabolized, mainly in the liver, into an active metabolite that is eliminated in the urine. The normal dose (5 mg once daily) should be reduced to 2.5 mg once daily in patients with moderate or severe renal impairment and excluded for patients with ESRD requiring hemodialysis 34. Meanwhile, linagliptin is the only DPP-4 inhibitor that is eliminated nearly entirely via the bile, thus making this agent a possible treatment choice for patients with normal kidney function as well as for patients in all stages of CKD, and even stage 5 (GFR <15 mL/min/1.73 m^2^), without dose adjustments 35. Alogliptin is primarily excreted unchanged in the urine and the usual dose is 25 mg once per day. However, for individuals with a decreased filtration rate, the dosage should be adjusted. Therefore, for patients with a creatinine clearance rate between 30 and 60 mL/min, the dose is 1.25 mg per day, whereas for those with greater loss of renal function or hemodialysis, the recommended dose is only 6.25 mg/day 36.

The safety of this therapeutic class has been questioned after the release of a phase 4 trial showing increase in hospitalization for HF. Certain risk factors are associated with this higher HF rate, such as previous HF, a GFR of <60 mL/ min and increased levels of N-terminal pro-B-type natriuretic peptide (BNP) 37. Moreover, a meta-analysis of 59 randomized controlled trials involving 36,620 patients treated with DPP-4 inhibitors for at least 24 weeks also showed a significant increase in the occurrence of HF compared with the placebo group. Furthermore, a potential interaction of the therapeutic class of gliptins with other drugs, such as angiotensin-converting enzyme inhibitors (ACEIs), BNP, substance P and neuropeptide Y (NPY), has been suggested 38.

### Incretin mimetics

Incretin mimetics include glucagon-like peptide 1 (GLP1) analogs and agonists (exenatide, lixisenatide and liraglutide), which increase insulin secretion and suppress glucagon secretion in a glucose-dependent manner, with reduced risk of hypoglycemia. These mimetics also slow gastric emptying and suppress appetite via central nervous system modulations, resulting in a reduced body weight. However, the main side effects are nausea and vomiting. Exenatide is predominantly eliminated by glomerular filtration and its clearance is mildly reduced in patients with mild or moderate renal impairment, whereas in subjects with ESRD, its removal is significantly reduced. Lixisenatide is eliminated through glomerular filtration, followed by tubular reabsorption and subsequent metabolic degradation, resulting in smaller peptides and amino acids that are introduced into protein metabolism. However, data from patients with kidney disease are limited. In persons without diabetes, mild-to-moderate renal impairment did not appear to affect the drug's pharmacokinetics or safety, but drug exposure was increased in persons with severe renal impairment, suggesting the need for dose adjustment in this population. Finally, liraglutide is metabolized in a manner similar to that for large proteins and its elimination is not related to a specific organ. Currently, its prescription in patients beyond mild-stage renal disease is limited and there are no recommendations that support its use in the moderate and severe stages 28,39.

### Sodium-glucose cotransporter 2 inhibitors

The sodium-glucose cotransporter 2 (SGLT2) protein, expressed in the kidney proximal tubules, is able to reabsorb approximately 90% of glucose filtered through the glomeruli. A new generation of drugs called SGLT2 inhibitors act by lowering plasma glucose by promoting glycosuria and osmotic diuresis, thus directly reducing plasma glucose concentrations in patients with hyperglycemia. This therapeutic class has been approved for the treatment of patients with T2DM with an eGFR of ≥45 mL/min/1.73 m^2^. To date, however, just canagliflozin has been evaluated, showing safety and efficacy in a subset of patients with stage 3 CKD 40,41.

Table 1 shows a summary correlating the therapeutic class and the dose adjustment based on creatinine clearance.

### Insulin therapy

The kidney also plays an important role in insulin clearance from the systemic circulation by distinct pathways. The first route involves glomerular filtration and subsequent absorption of insulin by proximal tubular cells through endocytosis. The second includes insulin diffusion through the peritubular capillaries and its binding to the contraluminal tubular membrane of cells and especially those located in the distal half of the nephron. As a result, insulin is transported by lysosomes and is metabolized into amino acids that are released into the peritubular vessels by diffusion, after which the final degradation products are reabsorbed 43,44. As exogenous insulin does not undergo a first-pass effect in the liver, the kidney plays an important role in removing insulin and is thus crucial to the metabolism and clearance of insulin in patients with renal impairment. CKD is associated with several disorders of insulin and carbohydrate metabolism and when renal failure advances, insulin clearance decreases, demanding a dose reduction to prevent hypoglycemia. This reduction in insulin clearance is initially compensated for an increase in insulin uptake by proximal tubular cells, and it is also associated with an increase in insulin resistance 45,46.

Few studies have examined the pharmacokinetics of long-acting insulin in diabetic patients with CKD. Although patients with impaired kidney function have lower insulin requirements, no dose adjustment is required if the GFR is >50 mL/min 45,47,48. Therefore, certain authors have suggested an insulin reduction to 75% of the total daily dose when the GFR is between 10 and 50 mL/min and to 50% for a GFR of <10 mL/min, independent of the insulin type being used 49.

Neutral protamine Hagedorn insulin (NPH) is known as an intermediate-acting insulin. Its time-action profile shows a typical peak within 4–7 hours after subcutaneous injection, and it is usually administered twice or three times daily 27. Insulin analogs, which are produced by recombinant DNA technology, could be short acting (lispro, aspart or glulisine insulin) or could be long- or ultra-long-acting basal insulin analogs (glargine, detemir and degludec). Used together, these analogs can simulate physiological insulin profiles more closely than regular and NPH insulin can 27,48.

Because of its pharmacokinetics and stable half-life, insulin glargine has a time-action profile of approximately 24 hours and should be prescribed as a once-daily medication 50. However, few studies have been published on the use of glargine insulin in patients with impaired renal function. One of these studies enrolled 89 patients with diabetes and a renal clearance of 34.1±11.5 mL/min who were treated with oral antidiabetic drugs or NPH insulin, although with sub-optimal glycemic control or frequent hypoglycemic episodes. Insulin treatment was then switched to insulin glargine at bedtime, with the addition of regular insulin to reach 100–140 mg/dL preprandial blood glucose. There was an improvement in glycemic control observed, based on the rapid decline in A1C (8.4±1.6% (68±4.5 mmol/mol) and 7.7±1.2% (61±5.3 mmol/mol) at baseline and after 4 months, respectively), showing safety and good tolerance for glargine insulin added to regular insulin in type 2 diabetes patients with diabetic nephropathy 51.

Another long-acting insulin analog that is commercially available is insulin detemir, which has an onset of action of 1 hour and a time-action profile of 12–24 hours, and, depending on patients' sensibility, it can be administered once daily 52,53. Kulozik and Hasslacher 48 showed that reduction in the GFR was associated with a lower dosage of two long-acting insulins (glargine and detemir). In other words, the insulin dosage at a GFR of <60 mL/min was 29.7% lower in glargine-treated patients and 27.3% lower in detemir-treated patients compared with the dosage at an eGFR of >90 mL/min. These authors did not find any difference between insulin detemir and insulin glargine with respect to sodium, albumin, or protein excretion in patients with type 2 diabetes and CKD.

A new-generation basal insulin with an ultra-long duration of action, insulin degludec, was approved to be commercialized, but there has been just one study in patients with different GFRs 54. This study evaluated thirty subjects with normal renal function; mild, moderate or severe renal impairment; or ESRD following a single subcutaneous dose of insulin degludec. No statistically significant differences in absorption or clearance, compared with subjects with normal renal function, were observed up to 120 hours post-dose, even in those with ESRD, regardless of whether the pharmacokinetic assessment period included hemodialysis. Based on this study, dose adjustments due to impaired renal function should not be required for insulin degludec 54.

Insulin lispro, insulin aspart and insulin glulisine are short-acting insulin analogs with very similar pharmacokinetic profiles. They are present either in monomeric or in very weakly bound hexameric forms, which are rapidly absorbed following subcutaneous injection. These insulin analogs usually reach their plasmatic concentration after approximately 60–90 minutes and have a duration of action of 3–4 hours 55. As insulin lispro was the first analog investigated, there have been several studies in patients with CKD 56-58. In type 2 diabetes patients with overt nephropathy, lispro insulin prevents glomerular hyperfiltration and reduces the renal effects of meal-associated hyperglycemia by mechanisms possibly related to insulin-like growth factor 1 (IGF-1) antagonism 56. Studies in a diabetic population with end stage renal disease undergoing hemodialysis treatment suggested that this analog can provide better glycemic control and improve quality of life 57,58. Insulin glulisine was also effective and safe in suppressing postprandial hyperglycemia in type 2 diabetes patients with severe renal insufficiency 59. Moreover, renal impairment does not affect the pharmacokinetics of insulin analogs in a clinically significant manner, as demonstrated for insulin aspart 60.

The search for the combination of keywords related to short- and long-acting insulin analogs yielded a few studies in patients with renal insufficiency. However, one study that enrolled hospitalized patients with the objective of evaluating insulin doses and hypoglycemia frequency showed that after initial reduction of glargine/glulisine insulin weight-based dosing, there was a notable decline in hypoglycemic events, or a 50% reduction, without compromising glycemic control 61. Regardless of the insulin considered as the best choice to improve glycemic control in patients with renal failure, specific information about dose adjustment and differences in insulin profiles in this population is still missing.

### Glycemic control in patients with diabetic kidney disease

Intensive glucose control decreases the risk of microalbuminuria and macroalbuminuria, but evidence is lacking as to whether intensive glycemic control reduces the risk of clinically significant renal outcomes, such as doubling of creatinine, ESRD or death from renal disease during the years of follow-up^61^. Many have tried to explain the controversy about the beneficial effect of tight glycemic control on clinically important renal endpoints. It appears difficult to undo both the changes secondary to a long-lasting high mean glucose level and the long-term effects on the development and progression of diabetes complications, even if tight glycemic control is maintained for many years afterward. A phenomenon known as “metabolic memory” or the “legacy effect” may be involved, including epigenetic programming, compositional changes, post-translational modifications, or simply lead time toward an inevitable fate 62.

Despite efforts to prevent morbidity and mortality in diabetes through guidelines and recommendations, a significant portion of the diabetic population still does not receive adequate care, as described by several reports auditing the quality of care 63,64. These studies' results demonstrate that adherence to guidelines is associated with better outcomes among patients with diabetes. However, adherence to practice guidelines is still low, especially regarding checking capillary blood glucose; performing eye examinations; achieving targets for blood pressure, A1C and low-density lipoprotein (LDL) cholesterol; and prescribing ACEIs and angiotensin II receptor blockers (ARBs), antiplatelet agents and statins. To address these controversies, the Standards of Medical Care in Diabetes 2014 of the American Diabetes Association (ADA) 8, the Kidney Disease: Improving Global Outcomes (KDIGO) 2012 guidelines 65, and the Clinical Practice Guidelines for the Evaluation and Management of Chronic Kidney Disease and the National Kidney Foundation Kidney Disease Outcomes Quality Initiative (KDOQI) guidelines for the management of diabetes with CKD 7 recommend a target A1C of <7.0% to prevent or retard the progression of CKD. Still, none of these guidelines defines the goals for glycemic control in patients with renal impairment and a GFR of <45 mL/min. In addition, several studies have questioned the accuracy of A1C as a reliable marker of the blood glycemic average, and extreme values for A1C, whether below 6% (42 mmol/mol) or above 9% (72 mmol/mol), have been correlated with the worst prognosis in individuals with significant loss of renal function 65,66.

The individualization of therapy, taking into account factors such as the presence of complications and other diseases, disease management capabilities, the CKD stage, the duration of disease, and previous glycemic control, among other factors, should be evaluated and may be considered as a safe and effective strategy for these patients 67-69. Therefore, a multidisciplinary team, including nephrologists, endocrinologists, dietitians and nurses, is important to help to identify individualized targets in patients with diabetic nephropathy. The health team is specifically responsible for supporting and motivating patients to follow a specific diet for diabetes control and renal protection, in addition to providing them with both orientation for exercise practice and explanations of the benefits of complying with the instructions, which is essential to prevent renal complications and/or to slow the progression of loss of kidney function.

The management of hyperglycemia in patients with DKD is even more difficult as a result of reduced glomerular filtration and its interference with glucose metabolism in many ways, favoring both hyperglycemic peaks and hypoglycemia. Additionally, renal failure impairs the clearance and metabolism of antidiabetic agents and insulin, thus frequently necessitating reassessment of prescriptions or adjustment of doses 48,70. Physicians responsible for the follow-up of these patients should be vigilant and prepared to make such changes; however, unfortunately, there are few guidelines addressing the nuances of the management of this specific population.

The dilemma of treating hyperglycemia in advanced kidney disease is how to balance tight glycemic control and harmful effects on the patient's safety. In other words, keeping glycemia within the normal narrow range to reduce the progression of the disease, improving quality of life, and minimizing comorbidities should be balanced, especially to avoid hypoglycemia, which is associated with increased cardiovascular risks. However, to date, the real benefits and impact of tight glycemic control in patients with long-standing diabetes and advanced CKD in particular are not yet fully known.

## AUTHOR CONTRIBUTIONS

Betônico CR conceived and designed the study and was responsible for the data acquisition. Titan SM, Correa-Giannela ML and Nery M were responsible for the data acquisition. Queiroz MS was responsible for the manuscript drafting, critical revision of the manuscript for important intellectual content and approval of the final version of the manuscript. All authors read and approved the final version of the manuscript.

## Figures and Tables

**Table 1:** Relationship among therapeutic class, medication dose and creatinine clearance.

Class and Medication	Dose Adjustment Based on eGFR
Biguanide	
Metformin	USA prescribing information: contraindication for men with serum creatinine ≥1.5 mg/dL and women with serum creatinine ≥1.4 mg/dL UK guideline allows metformin in patients with eGFR >30 mL/min/1.73 m^2^ KDIGO recommends metformin in patients with eGFR >45 mL/min/1.73 m^2^
Sulfonylureas	
Glipizide	No dose adjustment required
Glimepiride	Initiate conservatively at 1 mg daily Avoid use if eGFR <60 mL/min/1.73 m^2^
Gliclazide	Reduce dose if eGFR <30 mL/min/1.73 m^2^. Not recommended if eGFR <15 mL/min/1.73 m^2^
Glyburide or glibenclamide	Avoid use in patients with eGFR <60 mL/min/1.73 m^2^
Meglitinides	
Repaglinide	Initial dose of 0.5 mg before meals when eGFR <30 mL/min/1.73 m^2^
Nateglinide	Caution when used with eGFR <30 mL/min/1.73 m^2^. Initiate with 60 mg before meals
a-Glucosidase inhibitors	
Acarbose	Avoid if eGFR <30 mL/min/1.73 m^2^
Miglitol	Avoid if eGFR <30 mL/min/1.73 m^2^
TZDs	
Pioglitazone	No dose adjustment required. Use with caution in patients with CKD and hypervolemia
GLP-1 receptor agonists	
Exenatide	Avoid if eGFR <30 mL/min/1.73 m^2^. When eGFR between 30 and 50 mL/min/1.73 m^2^ dose should not exceed 5 mcg
Lixisenatide	Avoid if eGFR <50 mL/min/1.73 m^2^
Liraglutide	Avoid if eGFR <60 mL/min/1.73 m^2^
DPP-4 inhibitors	Sitagliptin and saxagliptin dose adjustment required based on eGFR
Sitagliptin	100 mg daily if eGFR <50 mL/min/1.73 m^2^
	50 mg daily if eGFR 30–50 mL/min/1.73 m^2^
	25 mg daily if eGFR <30 mL/min/1.73 m^2^
Saxagliptin	5 mg daily if eGFR <50 mL/min/1.73 m^2^
	2.5 mg daily if eGFR <50 mL/min/1.73 m^2^
Alogliptin	1.25 mg per day when eGFR 30–60 mL/min/1.73 m^2^, and for those patients with eGFR <30 mL/min/1.73 m^2^ or hemodialysis, the dose should not exceed 6.25 mg/day
Linagliptin	No dose adjustment required
	SGLT2 inhibitors	
Canagliflozin	No dose adjustment required if eGFR <60 mL/min/1.73 m^2^
	100 mg daily if eGFR 45–59 mL/min/1.73 m^2^
Dapagliflozin	Avoid use if eGFR <60 mL/min/1.73 m^2^, and discontinue use if eGFR <45 mL/min/1.73 m^2^

Legend: USA: United State of America; UK: United Kingdom; KDIGO: Kidney Disease Improving Global Outcomes; eGFR: estimated glomerular filtration rate; TZDs: thiazolidinediones; GLP-1 receptor agonists: glucagon-like peptide-1 receptor agonists; DPP-4 inhibitors: dipeptidyl peptidase 4 (DPP-4) inhibitors; SGLT2 inhibitors: sodium- glucose cotransporter 2 (SGLT2) inhibitors.
